# Oxidative-Stress-Sensitive microRNAs in UV-Promoted Development of Melanoma

**DOI:** 10.3390/cancers14133224

**Published:** 2022-06-30

**Authors:** Alessandra Pecorelli, Giuseppe Valacchi

**Affiliations:** 1 Department of Animal Science, N.C. Research Campus, Plants for Human Health Institute, North Carolina State University, Kannapolis, NC 28081, USA; apecore@ncsu.edu; 2 Department of Environment and Prevention, University of Ferrara, 44121 Ferrara, Italy; 3 Department of Food and Nutrition, Kyung Hee University, Seoul 02447, Korea

**Keywords:** skin cancer, sunlight, redox imbalance, miRNome, mutations, epigenome

## Abstract

**Simple Summary:**

Exposure to ultraviolet (UV) rays from the sun is one of the most important modifiable risk factors for skin cancer. Melanoma is the most life-threatening type of skin cancer. UV-induced DNA damage and oxidative stress represent two main mechanisms that, directly and indirectly, contribute to melanomagenesis. In addition, an interplay of abnormally expressed microRNAs (miRNAs) and redox imbalance is a hallmark in several cancers, including melanoma. UV radiation can be the central hub between these two cellular aberrations, as it is able to stimulate both. Here, to gain new mechanistic insights into melanomagenesis and identify new therapeutic targets for the prevention and treatment of melanoma, we report current evidence suggesting a complex interaction between UV-promoted deregulation of redox-sensitive miRNAs and known signal-transduction pathways underlying malignant transformation of melanocytes to melanoma.

**Abstract:**

Melanoma is the most aggressive and life-threatening form of skin cancer. Key molecular events underlying the melanocytic transformation into malignant melanoma mainly involve gene mutations in which exposure to ultraviolet (UV) radiation plays a prominent role. However, several aspects of UV-induced melanomagenesis remain to be explored. Interestingly, redox-mediated signaling and perturbed microRNA (miRNA) profiles appear to be interconnected contributing factors able to act synergistically in melanoma initiation and progression. Since UV radiation can promote both redox imbalance and miRNA dysregulation, a harmful crosstalk between these two key cellular networks, with UV as central hub among them, is likely to occur in skin tissue. Therefore, decoding the complex circuits that orchestrate the interaction of UV exposure, oxidative stress, and dysregulated miRNA profiling can provide a deep understanding of the molecular basis of the melanomagenesis process. Furthermore, these mechanistic insights into the reciprocal regulation between these systems could have relevant implications for future therapeutic approaches aimed at counteracting UV-induced redox and miRNome imbalances for the prevention and treatment of malignant melanoma. In this review, we illustrate current information on the intricate connection between UV-induced dysregulation of redox-sensitive miRNAs and well-known signaling pathways involved in the malignant transformation of normal melanocytes to malignant melanoma.

## 1. Introduction

Melanoma and nonmelanoma skin cancers represent the most common malignancies in white populations [1]. Basal cell carcinoma (BCC) and squamous cell carcinoma (SCC) are the prevalent forms of malignant skin cancers that develop from keratinocytes. Melanoma, which originates from melanocytes, is less common than keratinocyte skin cancers, accounting for only about 1–2% of all skin tumors, but it is the most aggressive and lethal type [1,2].

Over the past few decades, the overall rate of skin cancers has been growing worldwide, especially in fair-skinned populations [1,2]. The increased incidence can be related to more efficient and sensitivity diagnostic tools leading to an early detection combined with other factors including changes in individual and social behaviors—e.g., an increase in outdoor activities and different clothing style preferences—and a longer life expectancy accompanied by larger elderly populations [1,2]. In this context, the interaction between unprotected exposure to ultraviolet (UV) rays and genetic susceptibility represents the most important risk factor for skin cancers, as indicated by many epidemiological studies [3,4].

Melanoma results from malignant transformation of melanocytes, which are cells derived from the neural crest that are characterized by the ability to produce the pigment melanin. Melanoma incidence has been steadily increasing over the past few decades, and today it represents the fifth most commonly diagnosed malignancy in the United States. White individuals have a higher probability of developing melanoma than other racial/ethnic groups. In addition, the incidence rises with age, being frequently diagnosed among people aged 65–74, especially in men [5,6].

Melanoma can develop de novo or arise from pre-existing lesions such as congenital or acquired nevi. In addition, melanoma most often occurs on habitually sun-exposed sites of our skin, but it is also found in sun-protected areas including the palm and sole. It is more prone to appear on the trunk (chest and back) in males and on the lower legs in females. Moreover, less common melanoma subtypes can emerge from melanocytes residing in meninges, uvea, and mucosal membranes. Based on clinical and histological features, melanomas are classified into four main subtypes: superficial spreading melanoma, nodular melanoma, lentigo maligna melanoma, and acral lentiginous melanoma [7]. Two growth phases commonly characterize the development and progression of melanoma. During the initial radial growth phase, neoplastic melanocytes slowly grow horizontally within the epidermis and sometimes within the papillary dermis. In the vertical growth phase, transformed melanocytes proliferate vertically, invading the dermis and subcutaneous tissue and acquiring a metastatic phenotype [7].

Several signaling pathways have been associated with abnormal proliferation, growth, survival, migratory, and invasive properties of neoplastic melanocytes. In particular, among the underlying molecular aberrations characterizing melanoma and its etiology, the most frequent molecular changes involve genetic mutations of *CDKN2A*, *CCND1*, *CDK4*, *MITF*, *c-KIT* and *MC1R* genes; dysregulation of mitogen-activated protein kinase (MAPK) and phosphatidylinositol 3-kinase (PI3K)-Akt pathways; aberrant p53, STAT3, NRF2, NFκB, cadherin, and Wnt signaling pathways; and epigenetic alterations [8,9,10,11,12]. Ultimately, a complex and intricate connection between these aberrant signaling pathways and genetic abnormalities leads to a cascade of molecular events promoting uncontrolled melanocyte growth, proliferation, differentiation, migration and greater cell survival, resistance to apoptosis, invasion, and metastasis, which collectively promote tumorigenesis [8,9,10,11,12].

Important risk factors for the development of melanoma could fall under the concept of the exposome, representing the totality of exogenous exposures that individuals experience over the course of their lives, including geographic residence and pollutome [13,14,15]. In addition, host risk factors such as skin phototype or ethnicity, number of nevi (both congenital or acquired), genetic susceptibility, family history of melanoma, and immunosuppression can interact with environmental components to promote melanomagenesis [13,14,15]. Among the exposome components, certainly the exposure to UV radiation, particularly a history of intense intermittent sun exposure, represents the most important exogenous factor for the development of skin cancers, including melanoma [13,14,15].

## 2. Ultraviolet Radiation

UV rays are a form of electromagnetic radiation emitted from sunlight and a variety of artificial sources including tanning devices, some lasers, and several types of lamps (i.e., fluorescent, halogen, and incandescent lights). UV rays are categorized according to their wavelengths into UVA (320–400 nm), UVB (280–320 nm), and UVC (100–280 nm) (Table 1).

Depending on their energy level and the ability to remove or excite electrons in atoms or molecules, causing damage to living tissues, UV rays are divided into ionizing and nonionizing radiation. In this regard, higher-energy UVC rays, which have the shortest wavelengths, are extremely harmful to the skin and eyes. However, they are almost completely absorbed by the ozone layer in the stratosphere; therefore, the only potential detrimental health effect of UVC can arise from the exposure to some UVC lamps and lasers. Stratospheric ozone also protects us from most of the short wavelengths in the UVB band, while less energetic UVA radiation almost completely reaches the Earth’s surface without being absorbed by the atmosphere. Accordingly, solar UV rays reaching the ground level comprise approximately 95% UVA and 5% UVB (Table 1). In this context, it is worth mentioning that depletion of the stratospheric ozone layer due to global environmental changes is likely to have serious impacts on human health in terms of UV exposure within the near future [16]. Furthermore, the growing popularity of artificial tanning, especially among young women, represents another dangerous practice that can increase the risk of skin cancer, in particular when the first exposure is before the age of 35 years.

Concerning the biological effects on exposed human tissues, UVA and UVB rays exhibit both similar and specific attributes. In addition, another important difference is related to the acute (short-term) or chronic (long-term) health outcomes of the UV exposure [17]. All these aspects will be discussed in more detail in the next section. Lastly, although unprotected exposure to UV radiation can cause damage not only to the cutaneous tissue but also to the eyes and even modulate the activity of central nervous, endocrine, and immune systems [18], for the purpose of this review, we will focus on describing its harmful effects on the skin, particularly related to the increased chance of developing melanoma.

## 3. Beneficial and Adverse Health Effects of Sunlight

Sunlight is an essential prerequisite for life on Earth, providing necessary light and energy. However, the shortwave component of sunlight, namely the UV radiation, can have both beneficial and deleterious effects on humans and other living organisms, depending on a combination of different aspects including wavelength (UVA, UVB, or UVC), irradiation dose (intensity x duration), and size of the exposure [19,20].

Among the positive benefits associated with sunlight, especially UV light, the best known is the cutaneous synthesis of vitamin D that begins with the conversion of its precursor 7-dehydrocholesterol to previtamin D3 through a photochemical reaction triggered by UVB rays [21,22]. By regulating the expression of more than 1000 target genes, this essential nutrient and hormone is involved in multiple physiological functions, including support of bones, muscles, and the immune system. Moreover, vitamin D also may be implicated in reducing inflammation and infection rates and in preventing diseases such as some cancers, cardiovascular disease, diabetes, and mood disorders, as well as dermatological conditions [22,23,24,25].

In most cases, the positive effects of sunlight on human health are ascribed to vitamin D’s properties; however, it has become increasingly evident that regular solar exposure may be beneficial to human conditions through the action of additional mechanisms. They are related to the UV-stimulated production and release of mediators such as serotonin, endorphins, and melatonin, as well as antimicrobial peptides (AMPs), nitric oxide released from the cutaneous stores, and carbon monoxide from hemoglobin [22,25,26,27]. In this context, phototherapy approaches have been developed to mimic the benefits of sunlight exposure by using a controlled administration of UV radiation to treat skin conditions characterized by localized inflammation due to an overreaction of the immune system including psoriasis, vitiligo, severe eczema, atopic dermatitis, and mycosis fungoides [28].

While humans can benefit from regular exposure to physiological doses of UV light, on the other hand, intermittent intense (short-term) exposures together with chronic (long-term) exposures to UV radiation are usually associated with important adverse health effects. Short-term overexposure to UV is mainly linked to erythema, photodermatoses, tanning, photokeratitis, and photoconjunctivitis, as well as a local and systemic immunosuppressive effect with increased incidence and severity of infectious diseases [29,30,31,32,33,34]. Instead, chronic exposure to UV radiation promotes premature skin aging; increases the risk of skin cancer (melanoma and nonmelanoma); is responsible for cataracts, pterygium, and ocular melanoma; and finally, potentiates various autoimmune diseases and activates some viral diseases [17,20,32,34,35].

Interestingly, both the physiological and pathological changes induced by sunlight, and specifically by UV light, may be related to similar underlying molecular and cellular mechanisms, which will be detailed in the next section. What determines the switch from the appearance of physiological responses to the development of pathological manifestations is the extent of induction of these processes.

## 4. Molecular Mechanisms of UV Damage to Skin Tissue

As mentioned above, among the three different types of UV, the shorter-wavelength UVC rays could be the most harmful to living organisms. However, as they are blocked by the Earth’s ozone layer, their damaging effects on human health are only related to artificially created UVC used in specific applications such as, for example, in germicidal irradiation, or produced by artificial sources such as lasers and mercury lamps. Unlike UVC, natural UVB and UVA bands are not completely absorbed in the atmosphere and reach the Earth’s surface in a proportion of approximately 5% UVB and 95% UVA of the total UV energy. Moreover, another important source of UVA and UVB able to impact skin is represented by the growing trend in indoor tanning habits in combination with poor knowledge or awareness of the associated skin health risks.

The depth of penetration of the different UV rays into the skin depends on the wavelength, with the longest UVA rays able to penetrate much deeper into the skin than UVB and UVC (Table 1). Indeed, UVC light is barely able to penetrate the skin’s outermost layer, while UVB penetrates completely through the epidermis and marginally into the papillary dermis. Conversely, UVA affects the full thickness of the dermis, both the papillary and reticular layers, including the underlying subcutaneous tissue [18,36].

In general, UVA rays are considered less harmful than UVB due to their lower energy levels. Nevertheless, as mentioned above, they are more abundant in natural sunlight and more penetrating than UVB rays; therefore, UVA light is not exactly innocuous. In fact, thanks to their ability to penetrate into the dermis and induce damage to collagen and elastin, UVA rays are the main cause of skin photoaging, wrinkles, and loss of elasticity. On the other hand, UVB light is mainly associated with erythema, edema, immunosuppression, and skin cancer. Furthermore, based on recent observations, UVA rays have also been recognized as carcinogenic due to their immunosuppressive and mutagenic properties (Table 1) [37].

Regarding the molecular mechanisms of UV-induced injury, both UVA and UVB can produce adverse biological effects by targeting, directly and indirectly, cutaneous biomolecules such as DNA, proteins, and lipids (Table 1). In fact, the damage can result from a direct adsorption of UV photons by different macromolecules, which results in lesions that alter their structure and function [38,39,40,41,42]. In particular, DNA is one of the main UV chromophores in the cutaneous tissue, and its direct UV absorption leads to photochemical reactions with the formation of two major types of DNA lesions such as cyclobutane pyrimidine dimers (CPD) and pyrimidine-pyrimidone (6–4) photoproducts (6–4PP). Moreover, UV light is absorbed by other cutaneous non-DNA chromophores such as urocanic acid, melanin and its precursors, heme and bilirubin, porphyrins, amino acids (i.e., tryptophan, tyrosine, phenylalanine, histidine, and cysteine), and carotenoids. Photon absorption by these non-DNA chromophores changes their molecular structure and induces formation of photoexcited states able to transfer the excitation energy to other interacting molecules with the generation of free radicals, ROS, and other toxic photoproducts that, in a vicious circle, propagate the photochemical damage to DNA and the other macromolecules within the cutaneous tissue [38,39,40].

Moreover, UVB and mainly UVA radiation can also lead to an indirect oxidative-mediated damage of cutaneous macromolecules by stimulating ROS/RNS (reactive nitrogen species) production through enzymatic reactions catalyzed by enzymes such as NADPH oxidase, cyclooxygenase, and xanthine oxidase, or by the involvement of mitochondria [38,39,40]. When UV-stimulated ROS target DNA molecules, various types of oxidative DNA lesions are induced, including DNA single-strand breaks, DNA–protein crosslinks, and altered DNA bases. In particular, the oxidation of the guanine bases, which produces 8-oxo-7,8-dihydroguanine (8-oxoG), is the most abundant form of oxidative DNA damage. Furthermore, UV-induced ROS also attack other major biomolecules, causing protein oxidation and lipoperoxidation that compromise cellular ultrastructure and function [38,39,40]. In this regard, it is worth mentioning that melanocytes are more vulnerable to UV-mediated oxidative injury than other skin cells, since their specialized function, namely the melanin synthesis, is an energy-consuming process that itself contributes to generating a large amount of ROS.

Through these direct and indirect effects on the cutaneous biomolecules, UVA and UVB are able to induce a cascade of other molecular and cellular signaling interactions that include, among others, activation of transcription factors, altered gene expression, changes in the cell cycle, induction of inflammatory responses, cellular senescence, and apoptosis [17]. Furthermore, through bystander signaling mechanisms involving the release of microvesicles and exosomes, UV radiation can propagate dangerous molecular signals from the irradiated cells to neighboring nonhit cells through the extracellular space, causing further diffusion of harmful mediators able to fuel OxInflammatory phenomena into the cutaneous tissue [43,44,45,46].

Altogether, these UV-triggered molecular and cellular signaling events have a profound impact on the cutaneous tissue, as they are able to produce both physiological and pathological effects. Indeed, as previously stated, the same mechanisms stimulating, for example, the production of vitamin D and AMPs can also promote the development of premature skin aging and carcinogenesis [22,26,27].

## 5. UV-Induced Mutations Are Related to Melanoma Development

To counteract the noxious effect of outdoor stressors, including UV-induced skin damage, the cutaneous tissue is equipped with a complex network of protective mechanisms [47,48]. For instance, an elaborate antioxidant defense system ensures protection against UV-induced tissue OxInflammation [34,46,49]. In addition, skin cells activate various DNA-repair processes, cell-cycle checkpoints, cell-death pathways, and immune-surveillance mechanisms to prevent DNA damage and reduce the risk of genome instability [47,48,50]. However, in combination with a number of other host and exposome factors, including skin phenotype, genetic susceptibility, history of sunburn, and lifestyle, as well as geographical aspects such as altitude, latitude, urbanization, and the pollutome, UV’s damaging effects can overcome the cellular defense mechanisms and lead to tissue damage [13,14,15]. In this condition, UV-induced impairment of the cellular defense responses results in the accumulation of genetic mutations in cutaneous cells, with possible contribution to the development of skin cancers, including melanoma [13,51].

Over the last decades, the most recent genomic technologies have improved current knowledge of the main genetic alterations associated with cutaneous melanoma [52]. UV-induced damage can promote mutations in both oncogenes and tumor-suppressor genes. In general, gain-of-function mutations that activate specific oncogenes are considered causal events in the initiation of cutaneous melanoma, while loss-of-function mutations of tumor-suppressor genes have been implicated in the mechanisms underlying melanoma progression.

Mutations of the oncogenes *BRAF* and *NRAS* are the most frequent genetic alterations in cutaneous melanomas. *BRAF*-mutant melanomas are prevalent in body areas of intermittent sun exposure, while *NRAS*-mutant melanomas are mainly observed in chronic sun-damaged areas. Although the most common *BRAF* V600 mutation is not a typical UV-specific signature lesion, UV exposure is likely to play a key role in the onset of *BRAF*-mutant melanoma through genomic effects on other genes or by inducing noncanonical mutations, as reported by Laughery et al. and explained at the end of this section [48,53,54]. Moreover, mutations in *RAS* family proto-oncogenes also have been associated with cutaneous melanoma development following exposure to UV radiation, despite these mutations mostly lacking the typical UV signature [55,56].

Mutations in the oncogene *c-KIT* are more frequent in melanomas arising within skin with chronic sun-induced damage than in nonchronic sun-damaged skin [57,58]. Furthermore, Hodis et al. identified statistically significant mutations that resulted from C > T transitions in genes, such as in *RAC1*, *STK19*, and *PPP6C,* and therefore were directly attributable to UV-induced DNA damage, providing definitive evidence for the UV-mediated mutagenic role in melanoma pathogenesis [11].

The tumor-suppressor gene *PTEN* represents another frequently mutated gene in melanoma. As reported in a study by Wang et al. [59], UV played a direct role in induction of melanomas associated with *PTEN* mutations that, indeed, showed characteristic UV signature lesions at dipyrimidine sites. Another UV target gene involved in melanomas is *TP53*. In particular, in their study, Viros et al. demonstrated UV’s ability to accelerate *BRAF* (V600E)-driven melanomagenesis through the induction of UV signature mutations within the tumor-suppressor *TP53* gene [53,60].

In spite of this evidence, to date, the connection between UV exposure and melanoma risk is still matter of debate, especially as melanomas can also appear in non-sun-exposed areas of our body [61]. However, a mutational profile analysis of genomes associated with a broad spectrum of human neoplasia revealed that classical UV signature mutations showed the highest prevalence in the cutaneous melanoma when compared to all different mutational signatures, confirming that UV-mediated mutagenesis is a critical determinant for melanoma development [62].

Moreover, it is important to note that, in addition to the classical signatures, atypical UV photoproducts can also be responsible for observed noncanonical oncogenic lesions, even at lower frequencies, in the most common melanoma driver genes, including *BRAF* and *NRAS* [54]. In fact, a recent whole-genome sequencing analysis conducted in yeast repeatedly exposed to UV identified novel UV mutation signatures induced by the formation of atypical, but highly mutagenic, photoproducts that were present in human skin cancers such as *BRAF*- and *NRAS*-mutant melanomas [54].

## 6. UV-Induced Redox Imbalance Modulates Redox-Sensitive miRNAs

UV radiation plays a central role in the malignant transformation of melanin-producing melanocytes through different mechanisms. In fact, as a direct consequence of UV’s genotoxic effects, canonical and noncanonical DNA photoproducts compromise DNA integrity, leading to gene mutations and an increased risk of cancer development. Furthermore, UV can also participate in melanomagenesis via indirect oxidative mechanisms. Indeed, UV-mediated oxidative damage to DNA and other biomolecules, including proteins and membrane lipids, can affect multiple biological processes, which can also induce perturbation of oncogenes and tumor-suppressor genes [38,39,40,41,42].

Interestingly, growing evidence also suggests an involvement of UV in the regulation of cutaneous miRNA profiles in both physiological and pathological conditions [63,64,65,66,67,68]. This class of small noncoding RNAs with a length of about 19–24 nucleotides mainly function by regulating gene expression via post-transcriptional mechanisms that include translation inhibition and degradation of target messenger RNAs (mRNAs) [69]. As key modulators of gene expression, miRNAs are proven to shape the cellular proteome, affecting various biological signaling pathways including cellular development and differentiation, metabolism, proliferation, migration, and apoptosis/necrosis [69]. Furthermore, abnormal expression of miRNAs has been associated with age-related diseases and cancer, including melanoma, making miRNAs potential sensitive biomarkers for noninvasive diagnosis, as well as novel promising candidates for innovative therapeutic approaches [70,71].

As for other outdoor stressors, including ozone [15,72], UV radiation could regulate the expression of miRNAs via oxidative-stress-related processes [64,67,73]. Indeed, although via different mechanisms, both UV and atmospheric pollutants such as ozone, cigarette smoke, and particulate matter mainly disturb skin health by affecting the redox homeostasis of the tissue via ROS production [17,34,44,46,49,74]. In turn, ROS and their secondary byproducts, such as F_2_α-isoprostane, 4-hydroxynonenal, malondialdehyde, and acrolein, represent efficient cell-signaling molecules that are implicated in both physiological and pathophysiological signal transduction [74,75]. In this regard, recent evidence highlighted that redox-mediated mechanisms played a critical role in the modulation of miRNA pathways [76,77,78,79]. On the other hand, it is important to note that miRNA regulatory systems also can influence cellular redox homeostasis, making the crosstalk between oxidative stress and miRNA a novel pathway for the development of new therapeutic strategies. Indeed, this reciprocal connection has been associated with different human diseases, including carcinogenesis [76,77,78,79].

In particular, the expression levels of miRNAs can be either inhibited or induced by redox signals, with subsequent alterations of the regulated target genes and related signaling pathways [76,77,78,79]. The redox regulation of miRNA expression involves multiple molecular mechanisms. For instance, oxidative stress has been implicated in the processing of pre-miRNA transcripts to mature miRNAs by modulating the activity of key enzymes for miRNA biogenesis, such as Drosha and Dicer. In addition, many miRNAs are targets regulated by redox-sensitive transcription factors such as NFκB, c-Myc, p53, c-Jun, HIF-1α, and FOXO. Finally, oxidative mediators can also influence miRNA expression levels through epigenetic modifications involving an altered activity of DNA methyltransferases (DNMTs) and histone deacetylases (HDACs) [76,77,78,79].

## 7. UV-Dysregulated Redox-Sensitive miRNAs Involved in Melanoma Development

Based on the above evidence, in addition to specific genomic alterations, epigenetic modifications such as those linked to UV-induced deregulation of redox-sensitive miRNAs represent a further mechanism involved in cancer initiation and progression by affecting target genes linked to signaling pathways associated with melanomagenesis [66,73]. In this section, we will summarize some evidence demonstrating a role of UV-mediated redox mechanisms in the dysregulation of miRNAs involved in melanoma development and progression (Table 2).

MiR-22 is a miRNA that is upregulated in melanoma [80]. In UV-exposed cells, the upregulation of miR-22 is linked to the activation of the ATM protein (ataxia-telangiectasia mutated kinase) [66,81]. This serine/threonine kinase is activated in response to DNA damage, in particular to regulate the repair/processing of double-stranded DNA breaks; in addition, ATM can respond to other cellular stressors, including ROS [82,83]. Although DNA-repair processes, such as those regulated by ATM, protect genomic integrity and prevent carcinogenesis, however a persistent and chronic activation of ATM, such as that mediated by UV-stimulated ROS production, can paradoxically support tumor progression and metastasis, even promoting chemoresistance, radioresistance, and cell survival [84]. In this regard, increased expression levels of phosphorylated ATM at ser-1981, which is an activated form of ATM induced by ROS [85], were observed in melanoma samples, and also were associated with tumor progression toward a more aggressive and malignant phenotype with poor prognosis in patients [86]. The underlying mechanisms by which ATM activity promotes tumor progression are only partly elucidated [87]. A possible mechanism could implicate the induction of miR-22 that, in turn, inhibits UV-induced apoptotic cell death through the repression of PTEN expression [81].

Several studies have indicated the involvement of a dysregulated expression of miR-125b in melanoma. An array analysis of miRNAs in melanoma tissues showed a significant decrease in miR-125b expression in melanocytic lesions [88]. In addition, miR-125b expression levels were inversely correlated with the metastatic potential of melanoma, being further decreased in metastasizing melanoma compared to nonmetastasizing tumors, and were associated with a poor prognosis and shorter survival [89,90,91,92]. Using miR-125b mimic-transfected melanocytes, Kappelmann et al. showed that miR-125b controlled melanoma progression via direct downregulation of c-Jun protein expression, with consequent suppression of cellular proliferation [93]. Furthermore, ectopic expression of miR-125b in melanoma cell lines also induced an increase in senescence markers (i.e., senescence-associated β-galactosidase, p27, p21, and p53) and decreased proliferation [89]. Based on this evidence, miR-125b seems to act as a tumor suppressor in melanoma. Interestingly, a ROS-mediated repression of miR-125b has been implicated in the carcinogenesis process [76]. This inhibition appears to be dependent on the hypermethylation of the miR-125b promoter via ROS-induced recruitment of DNA methyltransferase 1 (DNMT1) to the sites of DNA damage [94]. Of note, UV-irradiated human skin also showed an overexpression of DNMT1 associated with an increased DNA hypermethylation of the TIMP2 promoter [95]. Therefore, this suggested that a redox mechanism promoted by UV light could contribute to melanoma progression by downregulating the tumor-suppressive miR-125b through the DNMT1-mediated hypermethylation of its promoter. In addition, other redox-responsive miRNAs were hypermethylated by oxidative mechanisms in melanoma; they included miR-9, miR-29c, miR-34b, miR-34c, miR-148, and miR-199a [96]. The epigenetic silencing of these miRNAs was associated with relevant oncogenic features of melanoma such as cell proliferation, migration, and motility. For instance, the promoter regions of the miR-199a gene also were hypermethylated under oxidative-stress conditions via a DNMT1-dependent mechanism [76]. Accordingly, low expression levels of miR-199a-5p (i.e., a miR-199 family member) were observed in melanoma tissue samples from patients in an advanced tumor stage [97]. In addition, transfection with miR-199a-5p mimics reduced cell proliferation in melanoma cells by decreasing HIF-1α expression, demonstrating the therapeutic potential of this and other miRNAs in melanoma [97]. Furthermore, decreased levels of the tumor suppressor miR-9 have been observed in melanoma tissues and correlated with enhanced methylation of its promoters [98,99]. UV could play a role in the epigenetic modification of this miRNA in melanoma cells, as already reported in UV-exposed keratinocytes, by inducing the methyltransferase DNMT3A [100].

In addition to epigenetic regulation, in the context of melanoma, a UV-induced redox imbalance could also modulate miRNA expressions via activation of transcription factors including NF-ĸB, c-Jun, p53, c-Myc, HIF-1α, and NRF2 [78]. In this regard, some evidence indicated an involvement of miR-206 in melanoma; for instance, its serum levels were decreased in patients with melanoma and were correlated with more severe clinical features, including higher number of metastatic sites, advanced stage, and poor response to treatment [101]. In fact, by targeting genes such as Cyclin C, Cyclin D1, and CDK4, miR-206 functions as a cell-cycle regulator and tumor suppressor in multiple melanoma cell lines [102]. Of note, in a mouse melanocyte cell line, three consecutive exposures to a low solar-simulated UV dose (60 mJ/cm^2^) triggered a simultaneous increase in cell proliferation with the modulation of the expression of several miRNAs, including miR-206 downregulation [66]. In melanoma, UV radiation could contribute to miR-206 modulation through a sustained activation of the redox-sensitive transcription factor NRF2 [103]. Although this endogenous defensive system plays an important defensive role, after tumor initiation and in response to elevated oxidative stress, it induced an altered cellular redox homeostasis, promoting uncontrolled growth and metastasis of melanoma [104]. These processes could be linked to the NRF2-dependent downregulation of the tumor suppressor miR-206, as observed in other cancer cells [103]. Other examples of redox-sensitive and NRF2-target miRNAs that are dysregulated in melanoma include the tumor-suppressive miR-200c and miR-193b [91,105,106]. Furthermore, NRF2, together with other redox-sensitive transcription factors such as SP-1 and NF-kB, are involved in a complex regulation of miR-29b, which also was implicated in melanoma [107].

During the transition from benign melanocytic lesions to malignant melanoma, an increased expression of the redox-sensitive miR-21 has been observed [108]. Interestingly, a number of exposome and host elements such as cigarette smoke, obesity, aging, chronic inflammation, and UV radiation, which represent major risk factors for melanoma, were associated with miR-21 upregulation [109]. In melanoma, overexpressed miR-21 promoted cell proliferation, migration, and invasiveness, as well as an increase in tumor cell survival and redox imbalance, by regulating target genes including, among others, PTEN, p53, cyclin D1, FOXO1, TIMP3, and HIF-1α [109]. The ability of UV to induce miR-21 overexpression in melanoma involves the ROS-mediated modulation of transcription factors such STAT3, AP-1, and NF-ĸB, which all have recognition sites on the miR-21 promoter [109,110]. In particular, UV rays could directly upregulate miR-21 expression in the melanocytic cells by activating STAT3 signaling [110]. Moreover, the uptake of miR-21-enriched exosomes from UV-irradiated keratinocytes could also contribute to further enhancing the total burden of miR-21 signaling in neighboring melanocytes via bystander effects [109,110]. In a reciprocal communication, a recent study also described the ability of UVA-irradiated melanocytes to induce enhanced proliferation and apoptosis resistance in surrounding keratinocytes via the release and transfer of extracellular vesicles. The STAT3/miR-21 axis was again the central hub of this intercellular crosstalk [111]. Moreover, miR-21 upregulation also was related to the UV-mediated ROS induction of NF-κB and AP-1 activities, two redox-responsive transcription factors that play important roles in UV-induced skin carcinogenesis [112]. In addition, other miRNAs dysregulated in melanoma and transcriptionally regulated by NFκB, possibly via an UV-dependent redox mechanism, included miR-9, miR-30b, miR-146a, and miR-155 [113,114,115].

Wnt/β-catenin signaling could be another redox-sensitive pathway involved in melanomagenesis through the induction of aberrant miR-182 expression. Overexpression of miR-182 in malignant melanoma tissues was associated with enhanced cell proliferation and invasiveness [116,117]. In other cancer and normal cell lines, miR-182 expression was controlled by β-catenin via redox-mediated events [118,119]. Of note, activation of β-catenin signaling pathway by UV light has been demonstrated in mouse melanocytes and human melanoma cell lines in vitro [120]. In addition, miR-182 was also among the miRNAs found to be upregulated in a study that evaluated the effects of a low solar-simulated UV dose on the miRNA profile of a mouse melanocyte cell line [66]. Finally, epigenetic mechanisms implicating CpG island methylation could also be involved in modulating the expression levels of miR-182 in melanoma [121].

## 8. Conclusions

Melanoma is the least common but the most aggressive and deadly form of skin cancer, especially in fair-skinned individuals [1,2]. It is a multifactorial condition arising from a synergistic interaction between different host and exposome factors, including genetic susceptibility and pollutome influence [3,4]. Among them, UV exposure is recognized as one of the most relevant modifiable risk factors involved in photocarcinogenesis [13,14]. In fact, although moderate sunlight exposure can produce positive effects in human health, minimizing prolonged unprotected UV exposure and the use of sunscreens are the key approaches to preventing skin cancers such as melanoma.

From a mechanistic perspective, genetic mutations are determinant drivers in the initiation, promotion, and progression of melanoma. Of note, the mutation frequency rate in melanoma is higher than that described for most other cancers. In this context, strong evidence indicates that UV-induced DNA lesions represent a major causative factor for mutations in melanoma-relevant genes [8,9,10,11,12]. Typical and atypical UV mutation signatures can be related to the direct absorption of UV energy with the formation of secondary photoproducts, as well as consequent to UV-generated ROS able to oxidize nucleotide bases [38,39,40,41,42].

In addition to promoting genomic instability, UV-related ROS can affect nearly all cellular processes, contributing to the generation of the heavily oxidized milieu of melanoma [38,39,40,41,42]. On the other hand, the critical role of ROS not only in skin carcinogenesis, but also in other cancers, is widely recognized [73]. In particular, UV-derived ROS and their secondary mediators can modulate melanoma-related transduction pathways, modify cell metabolism and gene expression, and cause epigenetic changes. Altogether, these alterations elicit molecular/cellular events that indirectly lead to cancer initiation and development [38,39,40]. Moreover, a new emerging hallmark of cancer pathogenesis is a detrimental crosstalk between ROS signaling and miRNA pathways [76,77,78,79]. Dysregulated miRNA profiling also appears to play an important role in melanomagenesis by altering the expression of several genes that interfere with biological processes such as cell growth and differentiation, metabolism, migration, and apoptosis/necrosis [71]. Since the cutaneous miRNAs profile is responsive to UV radiation through redox-dependent processes [63,64,65,66,67,68], it is conceivable that this UV-mediated dysregulation could be a further mechanism that promotes the development of the typical melanoma miRNome. As previously described, this UV-dependent redox regulation can implicate the interference with miRNA biogenesis and processing, the involvement of redox-sensitive transcription factors, as well as the induction of epigenetic modifiers [76,77,78,79].

Together, these findings suggested UV-mediated redox mechanisms as important regulators in the expression and function of miRNAs involved in melanoma initiation and progression. Considering that global warming and climate change are further altering the conditions of solar UV radiation on the Earth’s surface, increasing the risk of a range of health effects for humans [16], a deeper understanding of the correlation between miRNAs responsive to UV-induced oxidative stress and the melanomagenesis process becomes crucial. In fact, future investigations in this field will allow us to identify specific UV-dysregulated, redox-sensitive miRNAs as new players and useful targets for alternative approaches aimed at preventing and/or treating this life-threatening skin disease.

## Figures and Tables

**Table 1:** Different types of UV rays and their characteristics.

UV Radiation Spectrum	UVA	UVB	UVC
Wavelength	320–400 nm	280–320 nm	100–280 nm
Energy level	Lowest	Medium	Highest
Ozone layer absorption level	Not absorbed	Mostly absorbed	Completely absorbed
Percent reaching the ground	>95%	<5%	0%
Skin penetrance	Epidermis, dermis, and subcutaneous layer	Epidermis and marginally into the papillary dermis	* Uppermost, nonliving cornified layer of epidermis
Molecular cutaneous effects	ROS formation; indirect DNA damage (i.e., oxidized DNA bases such as 8-oxoG); protein and lipid oxidation	ROS formation; direct DNA damage (i.e., CPDs and 6–4 PPs); protein and lipid oxidation	Direct DNA damage (i.e., CPDs and 6–4 PPs); oxidative stress
Biological effects	Immediate tanning; sunburn; photoaging, wrinkles and loss of elasticity; some skin cancers	Delayed tanning; sunburn; erythema, edema, immunosuppression and skin cancer; premature aging	Redness; ulcers; skin cancer; premature aging

* UVC rays from artificial sources such as lasers and mercury lamps. Abbreviations: 6–4 PPs, 6–4 photoproducts; 8-oxoG, 8-oxo-7,8-dihydroguanine; CPDs, cyclobutane pyrimidine dimers; ROS, reactive oxygen species.

**Table 2:** Expression, mechanisms of regulation, and physiopathologic consequences of UV-induced, redox-sensitive miRNAs involved in melanomagenesis.

miRNAs	Expression	Mechanism of UV-Related ROS Regulation	Physiopathologic Changes
miR-22	↑ *	ATM phosphorylation/activation/PTEN repression	Decreased apoptosis; progression to metastatic phenotype
miR-9, miR-29c, miR-34b, miR-34c, miR-125b, miR-148, and miR-199a	↓ *	Promoter hypermethylation via DNMT activation	Increased cell proliferation, migration, and motility; progression to metastatic phenotype
miR-206, miR-200c, and miR-193b	↓	NRF2-dependent transcriptional regulation	Increased cell proliferation; progression to metastatic phenotype
miR-21	↑	STAT3-, AP-1-, and NF-kB-dependent transcriptional regulation	Increased cell proliferation, migration, and invasiveness, as well as increases in tumor cell survival and redox imbalance
miR-9, miR-30b, miR-146a, and miR-155	↑	NF-kB-dependent transcriptional regulation	Increased cell migration and invasion
miR-182	↑	Wnt/β-catenin-dependent transcriptional regulation	Increased cell proliferation, migration, and invasion; inhibition of cell apoptosis

* Symbols: ↑, upregulated expression; ↓, downregulated expression.
